# The regulation role of calcium channels in mammalian sperm function: a narrative review with a focus on humans and mice

**DOI:** 10.7717/peerj.18429

**Published:** 2024-10-25

**Authors:** Yebin Yang, Liu Yang, Xiaoqun Han, Kuaiying Wu, Guangquan Mei, Baojian Wu, Yimin Cheng

**Affiliations:** 1 Jiangxi Provincial Key Laboratory of Natural Active Pharmaceutical Constituents, Department of Chemistry and Bioengineering, Yichun University, Yichun, China; 2 Center for Translational Medicine, Department of Medicine, Yichun University, Yichun, China; 3 College of Traditional Chinese Medicine, Guangzhou University of Chinese Medicine, Guangzhou, China

**Keywords:** Calcium channels, Mammalian sperm function, Male infertility, Fertilization

## Abstract

Mammalian sperm are characterized as specialized cells, as their transcriptional and translational processes are largely inactive. Emerging researches indicate that Ca^2+^ serves as a crucial second messenger in the modulation of various sperm physiological processes, such as capacitation, hyperactivation, and the acrosome reaction. Specifically, sperm-specific calcium channels, including CatSper, voltage-gated calcium channels (VGCCs), store-operated calcium channels (SOCCs), and cyclic nucleotide-gated (CNG) channels, are implicated in the regulation of calcium signaling in mammalian sperm. Calcium stores located in the sperm acrosomes, along with the IP3 receptors in the neck of the redundant nuclear envelope and the mitochondria in the tail, play significant roles in modulating intracellular Ca^2+^ levels in sperm. However, the functions and mechanisms of these calcium channels in modulating mammalian sperm physiological functions have not yet been well elucidated. Therefore, by focusing on humans and mice, this study aims to provide a comprehensive review of the current advancements in research regarding the roles of calcium signaling and associated calcium channels in regulating sperm function. This endeavor seeks to enhance the understanding of calcium signaling in sperm regulation and to facilitate the development of drugs for the treatment of infertility or as non-hormonal male contraceptives.

## Introduction

The genome of mammalian sperm is primarily organized and compacted by sperm-specific nuclear proteins known as protamines (Ribas-Maynou et al., 2021). Protamines possess a high positive charge, which facilitates strong binding to sperm nuclear DNA (Balhorn, 1982). This protamine-DNA complex significantly contributes to the greater degree of DNA condensation observed in sperm compared to somatic cells (Ribas-Maynou et al., 2021). Such extensive DNA condensation renders mammalian sperm nearly transcriptionally and translationally inactive, with the exception of minimal protein translation occurring in the mitochondria (Gur & Breitbart, 2006). Consequently, the functions of sperm are predominantly regulated by post-translational processes (Camello-Almaraz et al., 2006; Gu et al., 2019).

At the moment of ejaculation, these cells rapidly exhibit high levels of progressive motility (Aitken & Nixon, 2013). Upon reaching the isthmic region of the oviduct, this behavior is abruptly reversed as the sperm establish intimate contact with the endosalpingeal epithelium. In this location, the adhered cells form a quiescent sperm reservoir, remaining in this state until they receive a signal associated with ovulation (Suarez & Pacey, 2006). During this process, a series of biochemical transformations occur, collectively referred to as capacitation. During capacitation, sperm develop a unique motility pattern known as hyper-activated motility, which ensures their ability to penetrate the viscous environment of the cervix (Suarez, 2008). As the sperm approach the oocyte, they are stimulated by various physiological factors secreted by the cumulus complex surrounding the oocyte (Redgrove et al., 2011). Subsequently, acrosomal enzymes released from the sperm’s acrosome facilitate penetration of the zona pellucida, ultimately leading to the successful fertilization of the oocyte (Inoue et al., 2011; Jin et al., 2011). Importantly, calcium signaling serves as a critical regulator of post-translational modifications, which in turn modulate the physiological functions of sperm (Li et al., 2020).

In mammalian sperm, various Ca^2+^-permeable ion channels, including CatSper (Orta et al., 2018; Wang et al., 2009; Chung et al., 2017, 2011; Hwang et al., 2019; Seifert et al., 2015; Navarro et al., 2008), voltage-gated calcium channels (VGCCs) (Lissabet et al., 2020; Albrizio et al., 2015; Wennemuth et al., 2000; José et al., 2010), store-operated calcium channels (SOCCs) (Nguyen et al., 2016; Krasznai et al., 2006), and cyclic nucleotide-gated (CNG) channels, play a crucial role in regulating intracellular calcium homeostasis (Fechner et al., 2015; Beltrán et al., 2014; Strünker et al., 2006). Notably, CatSper, a sperm-specific calcium channel, is essential for the primary influx of Ca^2+^ in mammalian sperm. This article elucidates the relationship between calcium signaling and sperm function, with a particular emphasis on the roles of various calcium channels in this process. Specifically, we review the function of CatSper channels in regulating male fertility in both mice and humans. This focus aims to enhance our understanding of the pathogenesis of male infertility and to identify specific targets for the development of non-hormonal male contraceptives. This review will appeal to scientists in the fields of andrology and reproductive medicine.

## Survey methodology

### Search strategy

Firstly, the databases searched in this study include PubMed, Google Scholar, Cochrane Library, and CNKI. The search terms are “calcium channels”; “mammalian sperm”; “CatSper”; “male infertility”; “voltage-gated calcium channels”; “store-operated calcium channels”; “cyclic nucleotide-gated channels”; “fertilization”; “sperm capacitation”; “sperm hyperactivation”; and “sperm acrosome reaction.” Secondly, the retrieval process is not limited by date or language, with the retrieval date set as June 31, 2024. A total of 457 articles were collected, primarily consisting of original research articles. Furthermore, the reference lists of primary articles were examined for additional relevant publications. Subsequently, the abstracts and main content of the articles were analyzed and screened for inclusion in the review.

### Inclusion and exclusion criteria

This study employed the following criteria to determine the inclusion of research: (1) a clearly defined timeframe for the studies; (2) specification of sample size to ensure generalizability and validity; (3) clarity in the criteria for sperm sample selection and processing, as well as intervention and control measures; (4) research that provides mean, standard deviation, and sample size for result quantification. The exclusion criteria included: (1) duplicate studies; (2) unreliable research data; (3) the use of incorrect statistical methods or instances where the experimental data did not indicate the mean and standard deviation.

## Effects of calcium signaling on mammalian sperm function

### Capacitation

In the testis, spermatozoa are derived from primordial germ cells and subsequently attain their fertilizing capability during their passage through the epididymis. The activation of sperm motility predominantly occurs at the time of ejaculation. Upon entry into the female reproductive tract, sperm undergo a process termed ‘capacitation’, which is essential for oocyte fertilization (Gervasi & Visconti, 2017). This capacitation process involves three Ca^2+^-mediated stages: initial activation, the initiation of a protein kinase cascade and hyperactivation of motility. When human spermatozoa are incubated in media that facilitate capacitation, a notable increase in protein kinase A (PKA) activity is observed within the initial 10 min. Conversely, processes such as protein tyrosine phosphorylation, cholesterol efflux, and the removal of decapacitation factors transpire over a prolonged period, spanning several hours (Stival et al., 2016).

These processes result in downstream physiological changes in sperm, specifically sperm hyperactivation, which facilitates detachment from the oviductal epithelium within the sperm reservoir. The increase in membrane fluidity facilitates lipid raft reorganization at the apical ridge regions and acrosomal remodeling, both of which are essential for acrosomal exocytosis (Sáez-Espinosa et al., 2020). In general, mammalian sperm capacitation is regulated by external and internal factors, primarily involving sterol acceptors (like albumin), bicarbonate, and calcium (Bailey, 2010; Macías-García et al., 2015). These molecules activate the protein kinase A (PKA) and protein tyrosine kinase (PTK) pathways, which are essential for capacitation. During the capacitation process, intracellular Ca^2+^ levels in mammalian sperm increase, primarily due to the activation of voltage-gated Ca^2+^ channels (Ca_V_), CatSper channels, and CNG channels (Darszon et al., 2011). Inhibition of these channels by utilizing antagonists can effectively impede the mammalian sperm capacitation process. The inhibition of these channels through the use of antagonists can effectively hinder the process of mammalian sperm capacitation. Moreover, an elevation in intracellular Ca^2+^ levels and pH_i_ within sperm cells can directly activate soluble adenylate cyclase (sAC), thereby promoting the production of intracellular cyclic adenosine monophosphate (cAMP). This, in turn, modulates the phosphorylation of sperm functional proteins *via* the cAMP/PKA pathway, and ultimately affect sperm capacitation (Vicente-Carrillo, Álvarez-Rodríguez & Rodríguez-Martínez, 2017; Alonso et al., 2017).

### Hyperactivation

In general, the initiation of sperm capacitation is inhibited within the male reproductive tract due to the presence of seminal plasma. Upon entry into the female reproductive tract, sperm undergo capacitation and acquire hyperactivated motility (HAM), which is characterized by high-amplitude flagellar beats and vigorous motility. This enhanced motility enables the sperm to pass the lumen filled with mucus with escaping the oviductal reservoir, bind to, and penetrate the zona pellucida, followed by fertilizing the oocyte after their arrival to the fertilizing site (Zaferani, Suarez & Abbaspourrad, 2021).

Bicarbonate, cAMP, and Ca^2+^ present in follicular fluids are recognized as pivotal elements in the induction of sperm hyperactivation. Both bicarbonate and cAMP promote the influx of extracellular Ca^2+^ by activating calcium channels in sperm, thereby establishing Ca^2+^ as an essential trigger for sperm hyperactivation in mammals. *In-vitro* studies have demonstrated that the presence of extracellular Ca^2+^ is crucial for the initiation of sperm hyperactivated motility (Bailey, 2010; Iwamatsu et al., 1985; Fraser, 1987). When hyperactivated hamster sperm were washed with a Ca^2+^-free medium, they maintained hyperactivation for 5 min but exhibited reduced motility after 30 min. The re-introduction of 1.8 mM Ca^2+^ in bath solution successfully restored hyperactivated motility (Iwamatsu et al., 1985). Furthermore, the Ca^2+^ ionophore A23187 transiently induces hyperactivated motility in boar and golden hamster sperm (Fraser, 1987). Notably, progesterone, which is prevalent throughout the female genital tract, facilitates extracellular calcium influx *via* activation of sperm-specific calcium channels (CatSper) (Lishko, Botchkina & Kirichok, 2011; Strünker et al., 2011). This process is critical for the induction of hyperactivated motility in sperm. Sperm deficient in CatSper channels are incapable of achieving hyperactivated motility post-capacitation, ultimately resulting in male infertility (Wennemuth et al., 2000; Darszon et al., 1999; Treviño et al., 2004; Lishko et al., 2012). In boar and bull, sperm hyperactivation can be activated by the influx of extracellular Ca^2+^. More significantly, akin to humans and mice, the expression of the CatSper channel protein is detectable in both boar and bull sperm. Concurrently, progesterone can activate the CatSper channels in these species, inducing hyperactivated sperm motility. Conversely, the CatSper inhibitors NNC 55-0396 and mibefradil markedly inhibit the progesterone-induced activation of sperm (Vicente-Carrillo, Álvarez-Rodríguez & Rodríguez-Martínez, 2017; Miki & Clapham, 2013; Romero-Aguirregomezcorta et al., 2019; Suarez et al., 1992; Holt & Fazeli, 2016; Song et al., 2011).

### Acrosome reaction

The acrosome, a structure resembling a cap situated in the anterior two-thirds of the sperm head, originates from Golgi vesicles. Enclosed within a membrane, the acrosome functions as a lysosome-like organelle housing various enzymes such as hyaluronidase, acrosin, acid phosphatase and so on. It is widely acknowledged that the acrosome reaction occurs when spermatozoa bind to the zona pellucida surrounding the oocyte. However, certain study propose that mouse sperm may not need to be acrosome-intact to facilitate this binding (Stival et al., 2016). In addition, it had been observed that rabbit sperm in the perivitelline space lacked acrosomes, yet these acrosome-reacted sperm could still penetrate the zona pellucida of other unfertilized oocytes (Kuzan, Fleming & Seidel, 1984).

During the acrosome reaction, the plasma membrane of the sperm fuses with the outer acrosomal membrane, facilitating the formation of a channel for the release of intra-acrosomal complex enzymes. Upon the completion of acrosomal exocytosis, the vesicles formed as a result of membrane fusion are entirely dispersed, resulting in the full exposure of the acrosomal matrix. This release enables the sperm cell recognize and bind to the ZP, ultimately fertilize the oocyte (Liu, Garrett & Baker, 2004; Liu et al., 2007; Abou-Haila & Tulsiani, 2000; Ramalho-Santos et al., 2001). Notably, the exposure of the acrosomal matrix is induced by an influx of extracellular calcium *via* channels, such as CatSper, store-operated Ca^2+^ channels and voltage-dependent Ca^2+^ channels (Gupta & Bhandari, 2011; Hirohashi & Yanagimachi, 2018).

In most mammalian except for murine and rabbit, the acrosomal exocytosis was induced upon contact with the ZP, and this process is primarily controlled by following signaling pathways (Inoue et al., 2011; Jin et al., 2011; Kuzan, Fleming & Seidel, 1984; Buffone, Hirohashi & Gerton, 2014). After the activation of receptors on the sperm surface such as ZP3R that are coupled with G-proteins, the Gi protein-coupled phospholipase C β1 (PLC β1) receptor initiates a signaling cascade that involves the activation of adenylyl cyclase (AC), leading to an increase in cyclic adenosine monophosphate (cAMP) levels in the cytosol (Breitbart, Rubinstein & Lax, 1997). This process further activates protein kinases and related proteins in the plasma membrane, ultimately facilitating the fusion of the plasma membrane with the outer acrosomal membrane of the sperm. Additionally, PLC cleaves phosphatidylinositol 4,5-bisphosphate (PIP2) into diacylglycerol (DAG) and inositol 1,4,5-trisphosphate (IP3). IP3 binds to IP3 receptors (IP3R) located in the acrosome and the redundant nuclear envelope (RNE) in the sperm’s neck region, which open IP3-gated channel and releases Ca^2+^ from internal store such as the acrosome or RNE into the cytoplasm (Walensky & Snyder, 1995). Upon the depletion of Ca^2+^ in intracellular stores, store-operated calcium channels (SOCCs) are activated. Furthermore, both DAG and Ca^2+^ can activate protein kinase C (PKC) (Gonzalez-Garcia et al., 2013), which will increase cytoplasmic calcium concentration by opening voltage-dependent calcium channel in plasma membrane. Ultimately, a sustained elevation in intracellular calcium concentration ([Ca^2+^]_i_) will promote the occurrence of the acrosome reaction (AR). In human sperm, the exchange protein directly activated by cAMP (EPAC), a Rap-specific guanine nucleotide exchange factor, has been demonstrated to mobilize Ca^2+^ from the sperm acrosome preceding the AR (Mata-Martínez et al., 2021).

## The role of calcium channels in modulating sperm function

### The sperm-specific calcium channel CatSper

#### The structure of the CatSper channel

CatSper, a sperm-specific calcium channel localized in the principal piece of mammalian sperm, comprises four primary subunits (CatSper1-4) and at least eight auxiliary subunits, including CatSperβ, CatSperγ, CatSperδ, CatSperε, CatSperζ, CatSperθ, EF-hand calcium binding domain 9 (EFCAB9) and C2 calcium dependent domain containing 6 (C2CD6) (Zhao et al., 2022; Yang et al., 2022). This multisubunit structure enables CatSper to respond to a variety of stimuli, including physiological factors such as progesterone and prostaglandins, as well as synthetic environmental compounds. Subsequently, CatSper is activated or inhibited by these endogenous or exogenous ligands, leading to changes in [Ca^2+^]_i_ that ultimately affect sperm physiological functions (Hwang & Chung, 2023).

Cryoelectronic analyses of mouse CatSper complexes have revealed that the central channel domain formed by CatSper1-4 adheres to the canonical architecture of voltage-gated ion channels (Jarow, 2002; Quill et al., 2001; Hwang et al., 2019). The selectivity filters of CatSper1-4, akin to those of Cav1.1, are protected by four critical residues: Asp536, Asp295, Asp227, and Asp237. Additionally, CatSper1-4 exhibit specific voltage-sensitive residues, including arginine or lysine in the S4 segments, charge transfer centers (CTCs), and aspartate or glutamate in the S2 segments (Lin et al., 2021). The cytosolic region comprises two distinct yet interconnected components: a subcomplex comprised of CatSper and EFCAB9 had been identified, while the component situated between elongated segments of CATSPER2 and CATSPER3 remains to be determined (Hwang et al., 2019).

The formation of a pavilion-like structure is achieved through the pairing of CATSPERβ, γ, δ, and ε with the S0 and S2 segments of CatSper4, 1, 3, and 2, respectively. Notably, CatSperδ is devoid of an N-terminal domain (NTD), a feature commonly found in the remaining three subunits. Subsequent to the NTD, all four subunits exhibit a structural composition consisting of a seven-bladed β-propeller domain, an Ig-like domain, and a stem domain preceding the transmembrane helix. Significantly, scientific investigators have determined that SLCO6C1, a protein responsible for organic anion transport and primarily expressed in the testis, interacts with CatSper through two interfaces. The glycosylation site and the residues implicated in the interaction between SLCO6C1 and CATSPER demonstrate a high degree of conservation across murine, rat, and human species (Lin et al., 2021).

#### The electrophysiological characteristics of CatSper in human and mouse sperm

When exposed to a divalent-free solution, CatSper channels predominantly facilitate the permeation of monovalent cations, including Na^+^ and Cs^+^ (Sun et al., 2017). The presence of Ca^2+^ on the extracellular side of the membrane led to a marked decrease in the monovalent current traversing CatSper channels. This finding suggests the presence of a high-affinity binding site for Ca^2+^ within the pore region of these channels, a feature typical of calcium-selective channels (Lishko et al., 2010). As the concentration of calcium ions in the bath solution was incrementally raised from a nominal level of 0 to 10 mM, while maintaining a pH of 8.0, a concomitant increase in the inward calcium current was observed. A comparable enhancement was observed when the concentration of Ba^2+^ in the bath solution increased from 0 to 50 mM, resulting in the generation of an inward current. Additionally, it was noted that the monovalent current, denoted as I_CatSper_, exhibited significant potentiation in response to intracellular alkalinization. Under physiological conditions, I_CatSper_ demonstrates a limited dependence on voltage, it is important to note that its voltage sensitivity is significantly influenced by the pH of the intracellular environment. Notably, the intracellular alkalinization that followed the administration of 10 mM NH4Cl further augmented the current through I_CatSper_. The use of mibefradil at a concentration of 30 µM resulted in a complete blockade of the monovalent current triggered by NH4Cl, the above findings strongly demonstrate that CatSper can be activated by intracellular alkalization (Strünker et al., 2011).

Progesterone is a steroid hormone released by cumulus cells surrounding the oocyte. At an intracellular pH of 6, the application of progesterone (1 μM) or NH4Cl (10 mM) gave rise to substantial barium tail currents. It was also observed that the simultaneous administration of NH4Cl and progesterone produced enhanced tail currents, exhibiting a non-additive interaction. The findings regarding the non-additive effects of changes in pH and the presence of progesterone, along with the inhibition by CatSper inhibitors NNC 55-0396 and mibefradil, confirm that alkaline pH, progesterone and PGE1 trigger the activation of CatSper channels. Similar to progesterone, PGE1 induced strong potentiation of monovalent I_CatSper_, and this activation could be fully suppressed by NNC 55-0396, therefore PGE1 can activate the CatSper channel in human sperm. More significantly, PGE1 and progesterone at saturation concentrations exhibit a synergistic effect on the activation of sperm CatSper channels, suggesting that progesterone and PGE1 facilitate Ca^2+^ entry into human spermatozoa *via* distinct binding sites. Conversely, no enhancement was observed in the mouse monovalent I_CatSper_ upon the addition of either progesterone or PGE1, likely reflecting the variation in the regulation of the primary sperm Ca^2+^ entry pathway among different species (Strünker et al., 2011).

#### The involvement of CatSper in modulating mammalian sperm function

##### Chemotaxis

The successful culmination of the fertilization procedure requires the sperm to navigate towards the ovum within the female reproductive system, a process that is regulated by at least three guiding mechanisms, namely chemotaxis, thermotaxis, and rheotaxis (Teves et al., 2009).

The function of CatSper in regulating mammalian sperm chemotaxis has been extensively documented, with evidence of both human and rabbit spermatozoa demonstrating sensitivity to picomolar concentrations of progesterone (Teves et al., 2006). Accordingly, progesterone, which is secreted by the cumulus cells that surround the oocyte, has been conclusively identified as a chemoattractant for sperm in humans. The exposure of human spermatozoa to progesterone gradients results in a progressive and temporary augmentation in intracellular calcium concentration ([Ca^2+^]_i_) within the sperm head, along with calcium oscillations at the base of the flagellum. These oscillations, elicited by low progesterone levels, modulate flagellar beat frequency without triggering the acrosome reaction. Apart from progesterone, chemotactic responses in mammalian sperm can also be induced by chemokine CCL20, natriuretic peptide type C (NPPC). However, the potential involvement of the CatSper channel in these processes remains to be clarified (Caballero-Campo et al., 2014; Kong et al., 2017).

##### Hyperactivation

Mammalian sperm acquire hyperactivated motility through an elevation in intracellular Ca^2+^ levels, which are derived from the extracellular environment. The necessity of extracellular Ca^2+^ for sperm hyperactivation directly implies the presence of Ca^2+^ channels on sperm, facilitating the influx of extracellular Ca^2+^ into the cytoplasm (Zhang et al., 2024). Various voltage-gated Ca^2+^ channels and transient receptor potential channels have been proposed as candidates for these Ca^2+^ channels involved in sperm hyperactivation. However, these channels have not been demonstrated to be the primary mediators of extracellular calcium influx in mammalian sperm. Currently, numerous studies suggest that mutations in the CatSper subunit can impair the development of hyperactivated motility in both murine and human sperm, ultimately resulting in male infertility. Consequently, CatSper is recognized as a principal Ca^2+^ channel essential for sperm hyperactivation, underscoring the physiological importance of the CatSper channel in male reproductive function (Ded et al., 2020; Avenarius et al., 2009; Schiffer et al., 2020; Avidan et al., 2003; Luo et al., 2019; Smith et al., 2013; Brown et al., 2018; Williams et al., 2015).

##### Acrosome reaction

The hypothesis that the CatSper channel is accountable for the initial transient increase of Ca^2+^ in sperm has been put forward. It is suggested that the influx of calcium ions into the sperm tail, mediated by CatSper, may result in an elevated concentration of calcium ions in the sperm head. This could potentially be accomplished by triggering a calcium ion-dependent release in the sperm neck. Nevertheless, the function of CatSper channels in the regulation of the acrosome reaction (AR) in mammalian sperm remains a contentious issue. In the context of mouse sperm, the propagation of calcium ions from tail to head does not appear to necessitate a CatSper-mediated increase in [Ca^2+^]_i_ (Pinto et al., 2023). In human sperm, the influence exerted by progesterone on the acrosome reaction is diminished when T-channel blocker NNC 55-0396 is present, which also inhibits the CatSper-mediated influx of calcium. These observations suggest that the CatSper channel could potentially govern the acrosome reaction in humans, however, additional studies are warranted to validate this hypothesis (Strünker et al., 2011).

#### Male infertility resulting from CatSper mutations in murine and humans

In mice, genetic disruption of any one of the four sperm-specific CatSper channels (CatSper1/2/3/4) leads to male infertility by impairing sperm motility (Chung et al., 2011; Jarow, 2002; Qi et al., 2007; Quill et al., 2003). For instance, studies conducted on mice have demonstrated that the absence of CatSper current and hyperactivated motility in the sperm of *CatSper1*^*−/−*^ mice leads to sterility. Specifically, in the sperm of *CatSper1*^*−/−*^ mice, the expression of CatSper primary subunits 2–4 and the accessory subunits (β, γ, δ and ε) was undetectable, indicating the critical role of CatSperβ, γ, δ, and ε in the assembly of the final channel complex in mature sperm. Subsequent functional analyses demonstrated that the sperm count and morphology of *CatSper1*^*−/−*^ mice did not significantly differ from those of wild-type mice (Jarow, 2002). However, notable reductions in sperm motility parameters, including path velocity, forward motility, and trajectory velocity, were observed in *CatSper1*^*−/−*^ mice. sperm in *CatSper1*^*−/−*^ mice exhibit symmetrical oscillations of low amplitude and frequency in a capacitation-conducing environment (Quill et al., 2003; Carlson et al., 2005).

This impaired motility prevents the sperm from generating the necessary driving force to detach from the epithelial tissue of the female reproductive tract and penetrate the zona pellucida of the oocyte. It is widely recognized that the abnormal hyperactivated motility observed in sperm deficient in CatSper1 is the primary factor contributing to their inability to fertilize the oocyte. Similarly, sperm from mice lacking the main CatSper subunits 2–4 do not exhibit hyperactivated motility in a capacitation-conducive environment, resulting in male infertility (Jin et al., 2007). Additionally, a separate study revealed that male mice deficient in the auxiliary subunit CatSperδ were sterile, with a notable decrease in the expression of the CatSper1 subunit, indicating CatSperδ and CatSper1 synergistically regulate sperm function with respect to structural integrity (Chung et al., 2011). Additionally, two auxiliary subunits of CatSper channels, CatSperζ and CatSperε, have been identified (Chung et al., 2017).

Notably, in contrast to deletions of other CatSper subunits, the infertility observed in CatSperζ^−/−^ male mice is predominantly due to a reduction in the oscillatory flexibility of the proximal flagellum. This reduction results in alterations in the three-dimensional flagellar envelope and impairs sperm motility within a simulated female reproductive tract environment (Jarow, 2002; Qi et al., 2007; Carlson et al., 2005).

Of note, a subsequent study conducted in 2023 provided a comprehensive examination of the crucial role played by the Tmem249-encoded transmembrane (TM) domain-containing protein, CatSperθ, in the formation of the CatSper channel during the evolution of sperm tail development. This study elucidated that CatSperθ is uniquely located at the intersection of a CatSper dimer. Male mice infertility is linked to the absence of *CatSperθ*, which removes the CatSper channel from the sperm flagellum, preventing sperm hyperactivation. It’s suggested that CatSperθ strengthens the CatSper4 subunit to build the CatSper complex and contributes to the formation of the necessary dimer for the channels’ zig-zag configuration (Huang et al., 2023).

In humans, male infertility related to *CATSPER* include both nonsyndromic male infertility (NSMI) and the deafness-infertility syndrome (DIS). The characteristic feature of syndromic male infertility is the presence of mutations in the genetic locus encompassing the *CATSPER2* gene, along with neighboring genes like *STRC* (stereocilin), which have been implicated in deafness. Meanwhile, NSMI is linked with mutations in the CATSPER1 and CATSPER3 subunits, which do not exhibit a correlation with deafness (Avenarius et al., 2009; Avidan et al., 2003; Zhang et al., 2009).

***CATSPER 2* Mutation**

The initial identification of syndromic male infertility (SMI) in relation to *CATSPER2* mutations was documented in a French family, where certain members exhibited a multifaceted phenotype encompassing infertility, deafness, and Congenital Dyserythropoietic Anemia type 1 (CDA1). Further observation revealed that all patients either partially or completely lacked the *CATSPER2* gene, presumably resulting in the absence of the CATSPER2 protein. A standard semen analysis conducted on two out of the three brothers in this family indicated normal semen volume and pH, yet revealed several sperm anomalies indicative of asthenozoospermia (Avidan et al., 2003).

The detected anomalies included a reduction in sperm count, decreased sperm motility with a percentage of progressive sperm falling beneath the standard threshold, compromised sperm viability, and a significant occurrence of abnormal sperm morphology. The most prominent sperm irregularities observed in these patients were coiled and angulated flagellum (Avidan et al., 2003).

In another instance, monozygotic twin brothers, aged 30, diagnosed with Deafness-Infertility Syndrome (DIS), were found to have a homozygous deletion of the STRC gene, resulting in the removal of the CATSPER 2 gene. A mutation in the CATSPER 2 gene has been associated with a decreased sensitivity to inducers of the acrosomal reaction and a complete inability for progesterone to activate intracellular calcium signaling within sperm (Nishio & Usami, 2022).

Further research has reported on three patients from an Iranian family, diagnosed with Congenital Dyserythropoietic Anemia Type I (CDAI), who also exhibited sensorineural deafness and a significant reduction in the percentage of motile sperm in semen. A comprehensive molecular examination of the DNA samples from this family revealed that the deletions at the 15q15.3 range span from 90 to 100 kb. These deletions invariably encompass the total loss of STRC and CATSPER2 at the genomic level. Consequently, the disruption of CATSPER2 could potentially be the underlying cause of significantly reduced sperm motility in patients suffering from infertility (Zhang et al., 2009).

It has been reported that the clinical phenotype in two patients from India, who possess a deletion in the 15q15.3 region, is distinct. A less severe manifestation of oligozoospermia is observed in a patient who has a single copy deletion of the *CATSPER2* gene, whereas a more severe phenotype is evident when both copies of the gene are deleted. Beyond infertility, no additional clinical phenotypes were identified in these subjects (Zhang et al., 2009).

A separate clinical instance of nonsyndromic male infertility (NSMI) was documented by a Chinese research team. They discovered a novel copy number variation (CNV) in *CATSPER2*, which disrupts the capacity of sperm to penetrate viscous media, undergo hyperactivation and respond to progesterone (Shu et al., 2015).

***CATSPER 1* Mutation**

Some scholars have identified two related Iranian families that exhibit nonsyndromic autosomal-recessive male infertility. In both families, insertion mutations were identified in exon 1 of *CATSPER1* (c. 539-540insT and c. 948-949insATGGC), resulting in frameshift and the generation of premature stop codons. The predicted mutant CATSPER1 proteins in both families lack all six transmembrane domains and the P loop. As a result, even if a truncated protein is synthesized, the CATSPER1 channel activity would be rendered ineffective in homozygous carriers of either mutation. It is important to note that the pH of the semen of both individuals was within the normal range. Nonetheless, the observed sperm were either nonmotile or exhibited motility below the standard threshold, accompanied by a decreased sperm count, an increase in abnormally structured sperm, and a reduction in semen volume (Avenarius et al., 2009).

A separate study enrolled 192 patients diagnosed with idiopathic asthenospermia and 288 healthy control subjects to elucidate the correlation between *CATSPER1* single nucleotide polymorphisms (SNPs) and idiopathic asthenospermia. Specifically, the exonal *CATSPER1* rs1893316 SNP, located in the first exon of the *CATSPER1* gene, demonstrated a significant correlation with the risk of idiopathic asthenospermia. The expression levels of both *CATSPER1* mRNA and protein were observed to be significantly diminished in patients possessing the TT genotype compared to controls and patients with the CC and CT genotypes. This suggests that the *CATSPER1* SNP (rs1893316) may play a role in the development of idiopathic asthenospermia by influencing the transcription of *CATSPER1* (Shu et al., 2015).

***CATSPER 3* Mutation**

A patient from a consanguineous family with non-syndromic male infertility was found to have a homozygous mutation in human *CATSPER3*. Intriguingly, despite the acrosome membrane remaining intact and the flagellum being normally assembled, the acrosome reaction was not induced by the calcium ionophore A23187 (5 μM). It is noteworthy that the favorable prognosis of this patient’s intracytoplasmic sperm injection (ICSI) treatment suggests that mutations in the *CATSPER3* gene may not adversely impact the maturity and integrity of the sperm nucleus (Zhang et al., 2009).

***CATSPERE* Mutation**

Upon conducting a thorough analysis of the DNA and sequencing data derived from a male patient previously diagnosed with a deficiency in the CatSper channel of sperm, investigators pinpointed a homozygous in-frame 6-bp deletion in exon 18 of *CATSPERE* (r761237686). This genetic aberration is anticipated to culminate in the elimination of two amino acids in the extracellular domain of CatSper-epsilon isoform 1. This type of modification may potentially instigate male infertility by impeding the function of the whole CatSper channel (Brown et al., 2018).

#### Factors involved in the activation of CatSper

##### Cytoplasmic alkalization

In most mammalian species, including rats, mice and humans, sperm are preserved in a quiescent and viable state within the cauda epididymis for a duration of several weeks prior to ejaculation. Upon release from the cauda epididymis during ejaculation, sperm demonstrate an immediate onset of vigorous motility (James et al., 2020). As they traverse the female reproductive tract, mammalian sperm encounter significant alterations in pH and ion concentrations in circumstances, which includes H^+^, HCO_3_^−^, Na^+^, K^+^, and Ca^2+^ (Ferreira et al., 2021). The N-terminus of CatSper 1 that rich in histidine (Kirichok, Navarro & Clapham, 2006), along with the EFCAB9-CatSperζ protein complex, enhance the sensitivity of CatSper to fluctuations in pH and Ca^2+^ levels (Hwang et al., 2019). Consequently, the CatSper channel is crucial for detecting these environmental alterations in female reproductive tract, which in turn modulate motility hyperactivation and potentially influence the acrosome reaction.

Generally, alterations in the cytoplasmic pH can influence sperm function by modulating CatSper channels. Studies reveal several mechanisms are involved in sperm pHi regulation and these mechanisms may vary across different species. In human sperm, intracellular pH is mainly be modulated by voltage-gated H^+^ Channel Hv1 and NHEs. It is noteworthy that the presence of Hv1 in human sperm had been verified by employing the membrane patch-clamp technique (Berger et al., 2017). Hv1 is directly activated by membrane depolarization, alkaline extracellular conditions, the endocannabinoid anandamide and the removal of extracellular zinc. Since both Hv1 and CatSper channels are located in the principal piece of the sperm flagellum, Hv1-mediated proton extrusion likely causes intra-cellular alkalinization. Specifically, when the intracellular pH was acidic (pH_i_ = 6.0), the voltage-dependent activation curves of CatSper channels exhibited a positive shift, suggesting that only a limited number of CatSper channels could open under normal membrane potential conditions. In contrast, an elevation of intracellular pH to 7.5 resulted in a leftward shift of activation curves, enabling the majority of CatSper channels to open at physiological membrane potentials. This cooperation between Hv1 and CatSper channels in human sperm may raise intracellular pH and Ca^2+^ levels, both of which are crucial for the regulation of sperm capacitation and hyperactivation in the female reproductive tract (Lishko & Kirichok, 2010).

Na^+^/H^+^ exchangers (NHEs) facilitate the exchange of Na^+^ and H^+^, which comprises 13 isoforms encoded by the SLC9 gene family. However, only three subtypes: NHE1, NHE5 and the sperm-specific NHE (sNHE) are present in sperm cells. A recent study revealed that 5-(N,N-dimethyl)-amiloride (DMA), a highly selective inhibitor of sodium/hydrogen exchangers (NHEs) that does not affect CatSper and KSper in both mouse and human sperm, adversely impacts the regulation of compartmentalized intracellular pH, the alkalization-induced activation of CatSper and KSper, and capacitation-associated functions in human sperm (Liang et al., 2024). These findings suggest that dysfunction in NHEs may contribute to the pathogenesis of male infertility. However, the specific NHE protein affected by DMA remains unclear, warranting further investigation.

Hv1 channels are additionally identified in bull sperm, where they are integral to the regulation of essential sperm functions, including hyperactivation, capacitation, and the acrosome reaction. These physiological processes are governed by a sophisticated interplay among cyclic adenosine monophosphate (cAMP), protein kinase C (PKC), and Catsper signaling pathways (Mishra et al., 2019). Conversely, Hv1 currents have not been detected in mouse sperm (Miller et al., 2015). Instead, Na^+^/H^+^ exchangers (NHEs) are posited to play a pivotal role in modulating the intracellular pH of mouse sperm (Kang et al., 2021). A sperm-specific Na^+^-H^+^ exchanger (sNHE) had been identified in murine, and mice lacking sNHE or exhibiting sNHE disruption demonstrate infertility attributed to impaired sperm motility (Wang et al., 2003). Similar to the Catsper channel, sNHE is situated in the principal piece of the sperm flagellum (Wang et al., 2007), indicating that the Catsper channel may respond to changes in intracellular pH (pH_i_) mediated by sNHE in mice.

##### Membrane potential

The primary determinant of membrane potential in both human and murine sperm is believed to be the permeability to potassium, which is regulated by Slo3, a constituent of the Slo family of potassium channels (Santi et al., 2010).

Preliminary patch-clamp recordings of CatSper currents have revealed that murine CatSper displays a relatively weak voltage-dependence with a slope factor (k) of 30, contrasting sharply with the markedly steeper k of approximately 4 noted in exclusively voltage-gated ion channels (Kirichok, Navarro & Clapham, 2006). Compared to mice, human CatSper has a steeper voltage dependence (k = 20) and a significantly higher half activation voltage (V_1/2_ human = +85 mV *vs* V_1/2_ mouse = +11 mV) at the same intracellular pH (7.5). Despite the CatSper channel exhibiting only a weak voltage dependence, alterations in membrane potential remain crucial for the optimal functioning of the channel, as they can impact not only the pH sensitivity of the CatSper channel, but also the activation of pH-regulating channels, such as Hv1 (Kirichok, Navarro & Clapham, 2006).

##### Endogenous and exogenous ligands of CatSper channel

Prior research has suggested that the CatSper channel is not only activated by intracellular pH and membrane potential, but also directly stimulated by a variety of endogenous ligands, including steroid hormones such as progesterone, prostaglandins, β-defensins and odorants (Lishko, Botchkina & Kirichok, 2011; Strünker et al., 2011; Brenker et al., 2012). These hormones are present in high concentrations in fallopian tube fluid and have been identified as potent activators of CatSper in human sperm (Carlson, Georg & Hawkinson, 2022).

Progesterone, a crucial endogenous ligand for human CatSper, promotes the hydrolysis of the CatSper channel blockade on the cytosolic membrane through the activation of the orphan enzyme α/β hydrolase domain-containing protein 2 (ABHD2) (Miller et al., 2016). Additionally, the influx of Ca^2+^ induced by P4 initiates several Ca^2+^-dependent physiological responses, such as hyperactivation, the acrosome reaction, and chemotaxis, all of which are vital for successful fertilization (Gonzalez-Garcia et al., 2013; Mata-Martínez et al., 2021). It is noteworthy that certain exogenous compounds, inclusive of plant triterpenoids (prestermelin, lupinol, et al.), endocrine disruptors (diethylestrol, PFOA, et al.), and pharmaceuticals (such as sertraline, mibefradil, RU1968F1, et al.) either inhibit or activate CatSper channels (Mannowetz, Miller & Lishko, 2017; Zou et al., 2017; Yuan et al., 2020; Rahban et al., 2021; Rennhack et al., 2018).

Synthesizing these findings, it can be inferred that CatSper functions as a polymodal chemosensor in mammalian sperm, which consequently positions it as an optimal target for selective male contraception.

##### Prospective non-hormonal pharmaceutical contraceptives for males

In the context of small-scale drug screening, HC056456, a non-steroidal compound, has been identified as a lead structure for a novel CatSper blocker. The study demonstrates that the CatSper current (I_CatSper_) induced by intracellular alkalization, can be reversibly inhibited by HC056456, thereby replicating the phenotype of CatSper deficiency in both HC056456-treated human and mouse sperm (Carlson et al., 2009). This inhibition results in impaired hyperactivation. Additionally, it has been observed that HC056456 has the potential to significantly diminish the success rate of *in vitro* fertilization (IVF) and entirely impede the sperm fertilization capacity in female mice that have undergone artificial insemination (Curci et al., 2021). Besides, RU1968, a potent and selective CatSper inhibitor, exhibited no cytotoxicity towards human and mouse sperm. It effectively inhibits CatSper activation induced by intracellular alkalization, progesterone and prostaglandin E1 (Rennhack et al., 2018).

Moreover, HC056456 and RU1968 have been found to inhibit other processes associated with sperm capacitation, such as tyrosine phosphorylation and progesterone-induced acrosome response. These findings suggest that the pharmacological blockade of CatSper channels by these drugs disrupts various Ca^2+^-dependent signaling pathways (Rennhack et al., 2018; Curci et al., 2021).

However, despite these findings, the propensity of HC056456 and RU1968 to obstruct other ion channels may result in unacceptable levels of toxicity. The recent identification of triazolopyridazine analogs, exhibiting robust CatSper-dependent current blockade and inhibitory activity on human sperm motility, is of significant interest. These analogs demonstrate a low affinity for ion channels associated with cardiotoxicity, including hCav1.2, hNav1.5, and hERG, and exhibit minimal cytotoxicity. These findings suggest that these analogs embody ideal pharmacological properties for potential application in male contraception (Carlson et al., 2022).

### Voltage-gated calcium channels (VGCCs)

**Figure 1:**
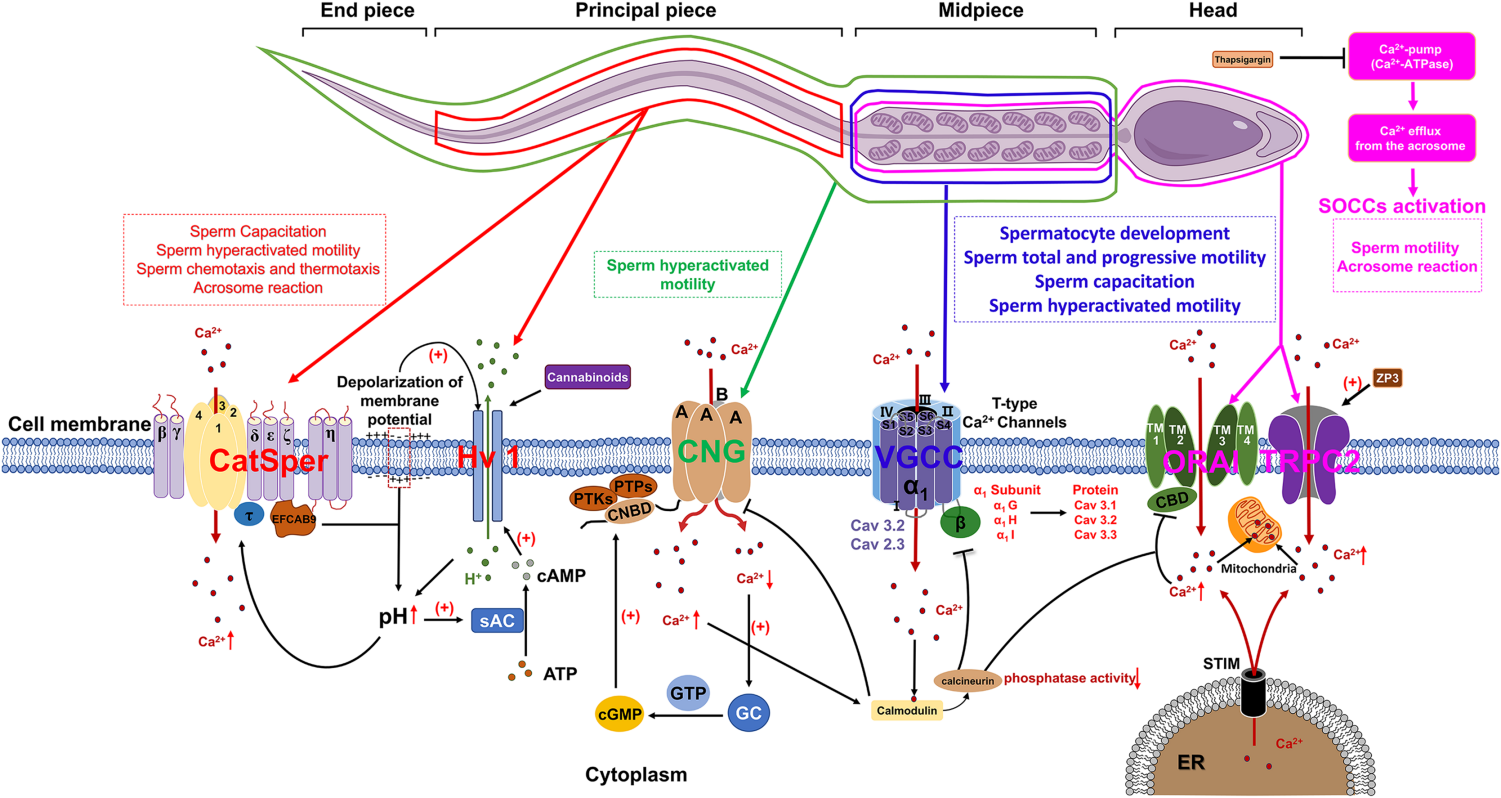
The role of calcium channels in the modulation of mammalian spermatozoa function. sAC, soluble adenylate cyclase; EFCAB9, EF-hand chiral calcium binding protein 9; CNG, Cyclic nucleotide-gated; PTKs, Protein tyrosine kinases; PTPs, Protein tyrosine phosphatases; CNBD, Cyclic nucleotide-binding domain; cGMP, Cyclic guanosine monophosphate; GC, Guanylate cyclase; VGCC, Voltage-gated Ca^2+^ channel; SOCCs, Store-operated calcium channels; ORAI, Orai1 channel; TRPC2, Transient receptor potential canonical channel 2; STIM, Stromal interaction molecule; ER, Endoplasmic reticulum; ZP3, Zona pellucida glycoprotein 3. Figure is drawn using a combination of PowerPoint and FigDraw platform (https://www.figdraw.com/). The sperm model is copied from https://www.figdraw.com/#/paint_img_info?clsId=undefined&pid=32778&nav=undefined&name=%E7%B2%BE%E5%AD%90, while the residual elements illustrated in the figure were produced utilizing PowerPoint.

Voltage-gated Ca^2+^ channels (VGCCs) constitute a class of transmembrane proteins. Under physiological conditions or at resting membrane potentials, VGCCs typically remain in a closed state. Membrane depolarization activates VGCCs, leading to Ca^2+^ influx, which elevates intracellular Ca^2+^ levels and subsequently initiates a cascade of alterations in cellular physiological processes (Hwang & Chung, 2023). In mammalian sperm, VGCCs are comprosed of α and β subunits, with the α subunits serving as the functional unit. Each α subunit contains four homologous transmembrane structural domains, each comprising six transmembrane hydrophobic α-helices (S1–S6). The voltage sensor is constituted by segments S1–S4, while the P-loop connecting segments S5 and S6 contains a conserved α-helix sequence and a glutamate residue responsible for ion selectivity (Fig. 1) (Yao, Gao & Yan, 2024).

VGCCs are categorized into three subfamilies: Cav1, Cav2, and Cav3. Cav1 and Cav2 are high-voltage-activated channels that require significant membrane depolarization for activation. These channels can be further classified into L-type, N-type, P/Q-type, R-type and T-type, based on their current characteristics. The *Cav3* gene encodes T-type calcium channels, which include the α1G (Cav3.1), α1H (Cav3.2), and α1I (Cav3.3) subunits. These channels are characterized by their activation at low membrane potentials and exhibit faster inactivation at more negative potentials compared to high voltage-activated (HVA) channels. The expression of these channels at protein level could be detected in both murine and human sperm (Fox, Nowycky & Tsien, 1987). Cav3.1 predominantly localized in the principal piece and mid-piece of the sperm flagellum. Additionally, Cav3.3 predominantly situated in the mid-piece of the flagellum in human sperm, which are similar to their localization in mouse sperm (Treviño et al., 2004). The functional regulation of Cav channels is crucial for the normal development of spermatocytes. The inhibition of either Cav1 or Cav3 channels by their respective blockers, nifedipine and ethosuximide, may lead to the disruption of normal spermatogenesis and steroidogenesis during prepubertal testicular maturation. These blockers are effective in inducing a premature arrest of developing spermatids and reducing Leydig cell abundance by inhibiting StAR protein expression, thereby diminishing testosterone production (Lee et al., 2011). During sperm capacitation, the influx of a small amount of Ca^2+^ through Cav3 channels into the cytoplasm significantly affects sperm physiological functions (Rehfeld, 2020). More importantly, Cav1 and Cav3 are additionally implicated in the regulation of the human sperm acrosome reaction (Wennemuth et al., 2000).

Previous research demonstrated that Ca^2+^ and calmodulin regulate the interaction between Cav3.2 and calcineurin. The binding of Cav3.2 to calcineurin results in a reduction of the enzyme’s phosphatase activity and subsequently decrease the current density of Cav3.2, which has been identified as a key regulator of sperm acrosome reaction (Camello-Almaraz et al., 2006; Lissabet et al., 2020; Albrizio et al., 2015). Furthermore, mice deficient in the α1E subunit of Cav2.3 exhibit significant subfertility in natural mating scenarios and demonstrate pronounced abnormalities in acrosome exocytosis (AE) and *in vitro* fertilization. These findings suggest that both Cav3.2 and Cav2.3 are involved in the regulation of acrosome reaction (AR) in murine sperm (Huang, Chen & Chen, 2013).

### Store-operated calcium channels (SOCCs)

In mammalian sperm, the release of Ca^2+^ from intracellular stores is frequently succeeded by the influx of extracellular Ca^2+^ through specific channels localized in the plasma membrane (Herrick et al., 2005). A plausible regulatory mechanism for this phenomenon involves the release of Ca^2+^ from intracellular stores, which induces the repositioning of the sensor stromal interaction molecule (STIM) protein, located on the membrane of the calcium store, into close proximity to the plasma membrane. This repositioning enables STIM to interact with store-operated calcium channels (SOCCs) and transient receptor potential canonical channels (TRPC), thereby facilitating the influx of extracellular Ca^2+^. SOCCs are composed of ORAI proteins (ORAI1-3), which possess four transmembrane segments (TMs) that form a pore between TM2 and TM3. Additionally, a Ca^2+^-binding domain (CBD) is located at the core of the pore (Fig. 1). This domain plays a crucial role in modulating channel function by interacting with either calcium ions or calmodulin (CaM), leading to the inhibition of SOCCs. Calcium ions entering the cytoplasm *via* SOCCs are stored in sperm mitochondria. Additionally, the distribution of STIM1, ORAI and TRPC have already been identified in mice and human sperm (Krasznai et al., 2006; Lefièvre et al., 2012; Darszon et al., 2012). STIM1 demonstrates a localization pattern analogous to that of the Ca^2+^ pool, being primarily concentrated in the acrosomal region, the neck, and the midpiece of the flagellum. The outer acrosomal membrane of sperm contains a Ca^2+^-pump (Ca^2+^-ATPase) (Schuh et al., 2004) and inhibition of this pump by thapsigargin results in a reduction of intra-acrosomal [Ca^2+^], subsequently triggering the activation of SOCCs (Parrish, Susko-Parrish & Graham, 1999). The influx of Ca^2+^ into the cytoplasm through SOCCs resulted in a sustained elevation of intracellular Ca^2+^ concentrations, subsequently triggering the acrosome reaction in mammalian sperm, encompassing species such as humans, mice, cattle, and goats (Lawson et al., 2007).

Currently, members of the TRPC family of transient receptor potentials are recognized as the most likely candidates for SOCCs in murine and human sperm (Castellano et al., 2003). The mRNA expression of TRPCs (TRPC1-7) could be detected in mouse spermatogonia, while the level of protein expression of TRPC1, 3, 6 proteins have been detected in mature mouse sperm (Trevino et al., 2001). Previous report proposed that TRPC2 may serve as a critical channel protein for sperm SOCCs. Immunohistochemical analyses revealed that TRPC2 is predominantly localized in the anterior region of the head, with a lesser extent in the posterior region of the head in mouse sperm. Upon stimulation by ZP3, TRPC2 emerged as a critical regulator of the sustained elevation in intracellular Ca^2+^ levels, consequently facilitating the acrosome reaction in murine spermatozoa. Subsequent investigations have demonstrated the extensive distribution of TRP channels in human spermatozoa, with expression localized to both the head and tail regions, thereby implicating a potential role in the modulation of human sperm motility (Jungnickel et al., 2001).

### Cyclic nucleotide-gated (CNG) channels

The cyclic nucleotide-gated (CNG) channel is constituted by four distinct A-type subunits, namely CNGA1, CNGA2, CNGA3, and CNGA4, as well as two B-type subunits, CNGB1 and CNGB3. Each subunit is typified by six transmembrane α-helices and a C-terminal cyclic nucleotide binding domain (CNBD) (Kaupp & Seifert, 2002). In mammalian, CNG channel is a heterotetramer that composed of A and B subunits, both of which belongs to the voltage-gated ion channel superfamily. Moreover, the functionality and permeability of cyclic nucleotide-gated (CNG) channels are modulated by cyclic guanosine monophosphate (cGMP) and cyclic adenosine monophosphate (cAMP). Genetic anomalies in the genes encoding CNG subunits can precipitate aberrant channel operation and instigate the emergence of clinical and pathological manifestations. Previous research has revealed disparate expression patterns of CNG subunits within the male reproductive system. In bovine, the B1 subunit expression is identifiable in bovine testis, with both the A3 and B1 subunits being distributed in the flagellum of mature bovine sperm. In murine, the B3 subunit is primarily expressed in the testis of mice and the A1 subunit is predominantly localized within spermatid remnants in the rat testis (Gerstner et al., 2000; Wiesner et al., 1998; Shi et al., 2005). Functional investigations have demonstrated that CNG channels in mouse sperm facilitate the influx of extracellular calcium, thereby triggering sperm capacitation (Cisneros-Mejorado & Sanchez Herrera, 2012). Notably, activity of CNG channel is mainly be regulated by intracellular cGMP or cAMP levels. The influence of cGMP on the permeability of CNG channels to Ca^2+^ was observed to be more substantial than cAMP.

In murine sperm, both protein tyrosine kinases (PTKs) and protein tyrosine phosphatases (PTPs) can phosphorylate serine residues S557 and S579 within the cGMP/cAMP-binding domain of CNG channels, facilitate the binding of cGMP to cyclic nucleotide-binding domain (CNBD) and subsequent modulate CNG channel permeability (Trudeau & Zagotta, 2003). Following the elevation of intracellular Ca^2+^ levels, calmodulin are activated, which suppresses the activity of cyclic nucleotide-gated (CNG) channels. Simultaneously, in order to maintain calcium homeostasis, the calcium pump Ca^2+^-ATPase isoform 4 (PMCA4) continues to export intracellular Ca^2+^ to the extracellular environment. The reduction of intracellular calcium levels induces the activation of guanylate cyclase (GC), which consequently amplifies the production of cyclic guanosine monophosphate (cGMP) as depicted in Fig. 1. This sequence of events subsequently stimulates CNG channels, thereby facilitating the preservation of calcium equilibrium between intracellular and extracellular compartments, which is a critical factor in the regulation of sperm motility (Darszon et al., 2008).

## Conclusion

The significance of calcium signaling in governing key physiological processes in mammalian sperm has garnered considerable interest. Although calcium channels located on the sperm plasma membrane are pivotal in regulating intracellular calcium signaling and related physiological functions, the precise functions and mechanisms of these identified calcium channels in governing sperm physiological processes are not yet fully elucidated. The continuous advancements in molecular biology and electrophysiological methodologies underscore the imperative for further investigation into the functions and mechanisms of both characterized and uncharacterized sperm calcium channels. This review seeks to enhance our understanding of male infertility’s causes and identify targets for non-hormonal male contraceptive development.

## Additional Information and Declarations

### Data Availability

The following information was supplied regarding data availability: This is a literature review.
